# Reflections on the Morbid Phenomena Caused by the Use of Iodine

**Published:** 1830-04-01

**Authors:** 


### LVIII.

### Reflections on the Morbid Phenomena Caused by the Use of Iodine. By Dr. Jahn.

The author of this paper, published in the Journal Complem. for Feb. 1830, informs us that in the part of country where he resides, there prevails a species of bronchocele which, to all appearance, is a simple hypertrophy of the thyroid gland. He affirms that this affection is never seen where the water springs from ground that is basaltic, porphyritic, granitic, or gravelly; whereas it is constantly observed where the waters issue from calcareous soils, and contain a quantity of lime in mixture. Iodine is the remedy which is generally employed for bronchocele in this part of the country, and he has had ample opportunities, he says, for remarking its effects on the animal organization.—Orfila shewed that this powerful medicine, in lagre doses, occasioned pains in the stomach, malaise, vomiting, diarrhœa, syncope, oppression, ptyalism, tremors, &c. Symptoms of this kind were observed by our author in a man who had swallowed, by mistake, an inordinate dose of the tincture of iodine. In other cases where the medicine was merely continued longer than proper, Dr. J. was struck with the slow but great absorption of fat, preceded and accompanied by an augmentation of all the secretions and excretions. He observed the skin became dark-coloured, and the perspiration viscid—the breathing embarrased—the urine abundant—the bowels loose and very bilious—the catamenia (in females) more copious. From these phenomena our author argues that the functions of the venous and absorbent vessels are much increased, in proportion as the nutritive system has been diminished. The blood, he says, becomes thinner than natural, and less abundant in fibrine. If the medicine be continued, in these cases, various bad consequences ensue. He mentions two cases where he opened the bodies of patients who laboured under the excessive use of iodine, and where the emaciation was extreme, and the viscera in a very flabby state. Still Dr. J. considers the medicine in question as one of the most valuable which has been discovered within the last hundred years. Indeed he terms it "un remede divin."

In scirrhus of the pylorus Dr. J. affirms that iodine has sometimes removed the malady, when early applied, and when aided by leeches. The same has been stated by Wagner, Henneman, Hufeland, and others. We would strongly recommend the medicine in question to be tried in dropsical cases dependent on visceral obstructions.

